# A microfluidic platform for trapping, releasing and super-resolution imaging of single cells

**DOI:** 10.1016/j.snb.2016.03.131

**Published:** 2016-09

**Authors:** Ying Zhou, Srinjan Basu, Kai J. Wohlfahrt, Steven F. Lee, David Klenerman, Ernest D. Laue, Ashwin A. Seshia

**Affiliations:** a Nanoscience Centre, Department of Engineering, University of Cambridge, 11 JJ Thomson Avenue, Cambridge CB3 0FF, United Kingdom; b Department of Biochemistry, University of Cambridge, 80 Tennis Court Road, Cambridge CB2 1GA, United Kingdom; c Department of Chemistry, University of Cambridge, Lensfield Road, Cambridge CB2 1EW, United Kingdom

**Keywords:** Hydrodynamic trapping, Particle manipulation, Single cell analysis, Super-resolution imaging, Embryonic stem cells

## Abstract

A multi-layer device, combining hydrodynamic trapping with microfluidic valving techniques, has been developed for on-chip manipulation and imaging of single cells and particles. Such a device contains a flow layer with trapping channels to capture single particles or cells and a control layer with valve channels to selectively control the trap and release processes. Particles and cells have been successfully trapped and released using the proposed device. The device enables the trapping of single particles with a trapping efficiency of greater than 95%, and allows for single particles and cells to be trapped, released and manipulated by simply controlling corresponding valves. Moreover, the trap and release processes are found to be compatible with biological samples like cells. Our device allows stable immobilisation of large numbers of single cells in a few minutes, significantly easing the experiment setup for single-cell characterisation and offering a stable platform for both single-molecule and super-resolution imaging. Proof-of-concept super- resolution imaging experiments with mouse embryonic stem cells (mESCs) have been conducted by exploiting super-resolution photoactivated localisation microscopy (PALM). Cells and nuclei were stably trapped and imaged. Centromeres of ∼200 nm size could be identified with a localisation precision of <15 nm.

## Highlights

• Efficient, reliable and long-term trapping of single particles and cells.
• Selective releasing or retrieving trapped particles/cells.
• A stable platform for super-resolution imaging at a near molecular resolution.
• Study of mouse embryonic stem cells using photoactivated localisation microscopy.
• Identify centromeres of ∼200 nm size with a precision of <15 nm.

## 1 Introduction

Super-resolution fluorescence microscopy approaches such as photoactivated localization microscopy (PALM) not only allows the visualisation of subcellular structures at a spatial resolution approaching that of biomolecules, but also allows the study of protein dynamics within individual cells [1]. They allow the 3D imaging of fluorescent proteins in cells with an accuracy far beyond the diffraction limit of visible light. Such super-resolution techniques for visualising tagged molecules within cells place strict requirements upon sample preparation and require target cells to be immobilised over an extended period of time for the highly precise imaging process [2] . For this purpose, long-term single cell trapping is necessary.

Due to the capabilities of precisely controlling biological samples, microfluidic systems provide unique opportunities for single cell study. Trapping of single particles and cells has been demonstrated recently in various microfluidic devices [3], [4], [5], [6], [7], [8]. Among various cell capture techniques, hydrodynamic trapping relies on a relatively simple fabrication process (e.g., soft lithography) and ease of implementation (no external instrumentation is required such as lasers, transducers or signal generators). Bell et al. have reported such a hydrodynamic trapping device for immobilisation of Schizosaccharomyces pombe cells to facilitate cell super- resolution microscopy. This device enables cell trapping and imaging, but it does not allow the trapped cells to be released or retrieved from the trapping site in a controllable manner. On the other hand, Tan et al. developed a hydrodynamic approach to reliably immobilise large numbers of beads and an optical-based technique for retrieving beads [9], [10]. The trapping process is based on hydrodynamic trapping and the differential fluidic resistance exhibited between the main and bypass channels in the device. Immobilised beads are released by heating the aluminium pattern near the trap with an infrared laser, thereby generating a bubble near the trap to displace the bead into the main flow. However, this release method requires beads being released in a specific order i.e., the bead upstream has to be released first and that located downstream needs to be released last, in order to avoid beads being re-trapped in empty traps downstream. In addition, there are concerns when using such method to release biological cells as the heat generated in the channel may result in harmful effects. Therefore, there is great interest in exploring bio-compatible approaches to trap and release particles or cells in a controllable manner. Multilayer microfluidic valving techniques may serve as a solution to this.

Microfabricated valves based on multilayer soft lithography were first described by Unger et al. [11] and have been widely used since then [12], [13], [14], [15]. A basic microfluidic valve contains two layers: one layer has the fluid to be controlled (flow layer); the other layer contains control channels (control layer). A valve is created when a control channel and a flow channel cross. When the control channel is pressurised, the valve membrane deflects into the flow channel and thus diminishes the size of the flow channel. The flow channel can be completely sealed when sufficient pressure is applied to overcome the elasticity of the elastomer membrane and the fluid pressure in flow layer (back pressure). When the pressure in the control channel is relieved, the elasticity of elastomer causes the valve membrane to spring back to its original position, opening the valve [16].

In this work, we present a microfluidics-based platform for super-resolution PALM imaging of single protein molecules in individual cells. We have combined hydrodynamic trapping with microfluidic valving techniques to accomplish single particle/cell trapping and release. The device described in this work not only enables single cell trapping with high efficiency, but also allows us to release and retrieve cells from the trapping sites in a controllable manner by controlling on-chip valves. Polystyrene particles with different sizes have been used to test the trapping efficiency of devices with different dimensions. The proposed trapping device is able to reliably and automatically capture and hold a large array of cells with a suitably high density, and thus, can be adapted to build a robust and user-friendly platform for super-resolution microscopy. Proof-of-concept experiments with mouse embryonic stem cells (mESCs) have also been conducted to demonstrate applicability as a research tool for single cell handling and super-resolution imaging.

## 2 Device design

**Fig. 1:**
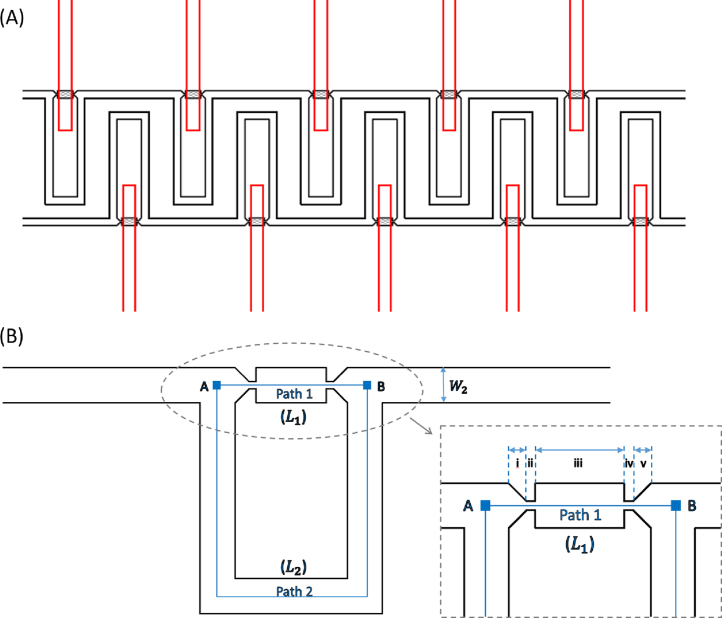
(A) Key layout features of a two-layer device. The black layer is the flow layer (containing trapping channels) where particle/cell trapping is accomplished. The red layer is the control layer (containing valve channels). The crossed area between the two layers forms a very thin PDMS membrane, which controls the opening or closing the flow channel, depending on the applied pressure in the control channel. (B) Schematic diagram showing the trapping channel in the flow layer. Inset shows a close-up view of Path 1, which contains five regions (notated as i–v respectively). (For interpretation of the references to colour in this figure legend, the reader is referred to the web version of this article.)

The device contains two layers: the flow layer (containing trapping channels) and the control layer (containing valve channels). The schematic diagram of the two-layer device is shown in Fig. 1A. The flow layer (black) contains an array of trapping sites for single particle/cell trapping. In each trapping unit, there are two opposite facing traps (for particle trapping in both directions). Between the two traps is a middle chamber. The particle/cell capturing process is based on hydrodynamic trapping and the differential fluidic resistance concept. The valve channel in the control layer (red) is right above and crossing the middle chamber in the flow layer, creating an active valve at the crossed area. The crossed area between the two layers forms a very thin PDMS layer (i.e., valve membrane), which controls the opening or closing the flow channel, depending on the applied pressure in the control channel. The microfluidic valve can be activated by pressurising the valve channel in the control layer and deactivated by relieving the pressure in the control layer.

When the middle chambers are open (valve is deactivated), single particles/cells can be efficiently trapped as normal. Once the particles/cells are trapped, the release process can be initiated by activating the valves and closing the middle chambers in the flow layer. There are two main factors that facilitates the release process: (1) Once the valve is activated, the valve membrane deflects downwards into the middle chambers in the flow layer, pushing the fluid out of the middle chamber. This force also pushes the trapped particles/cells out of the trapping site. In the meantime, valve membrane deflecting into the flow channel diminishes the size of the flow path and changes the flow resistance, further facilitating the particle/cell release process. (2) Once the middle chamber is fully sealed by the valve membrane, the flow via the short and straight trapping path will completely stop. The only flow path remaining in the flow channel that particles/cells can follow is via the bypass loop channel. Particles and cells will be driven into the main flow by the drag force of the fluid and thus be released from the traps.

If all the traps are controlled by a common valve (Fig. S1A in Supplementary material), high throughput particle/cell trapping and release could be done by simply controlling the common valve. On the other hand, if each trap is controlled by individual valves (Fig. S1B), selectively trapping and releasing the particles/cells would be possible by activating or deactivating the corresponding valve located on top of each middle chamber. This aspect of the design is useful for single cell culturing and characterisation, as single cells can be trapped at desired positions for real-time and long-term analysis, after which the cells that are of interest can be selectively released and retrieved from the device for further processing.

Fig. 1B is a schematic drawing of a trapping unit. There are two paths from point A to point B: Path 1 and Path 2. The straight channel, Path 1, can be subdivided into five regions (notated as i–v), as shown in the inset of Fig. 1B. Region i and v are the actual positions where cells will be physically trapped, so these two regions are referred to as “traps”. These two traps are facing oppositely. Depending on the flow direction, one of them will be occupied while the other one will be empty. The narrowest regions in Path 1 (ii and iv), known as the trapping gaps, are smaller than cells and thus allow cells to be mechanically stopped and immobilised tightly in place in the traps (region i and v). Path 2 is a bypass channel, which shunts cells away from an occupied trap and leads them to the next one. The flow resistance along Path 1 is designed to be lower than that of Path 2, so that a particle can be driven into a trap by hydrodynamic forces when the trap is empty [9], [10]. Once the trap is occupied by a cell, the flow through Path 1 is blocked, and thus the next cell will be driven into the bypass channel and enter the next available trap.

The width and total length of the bypass channel (i.e., Path 2) are indicated as *W_2_* and *L_2_*, respectively. The total length of Path 1 is *L_1_*, and Path 1 is composed of five regions (indicated as i–v, in Fig. 1B). Each region in Path 1 has its own dimensions such as width and length: *W_11_* and *L_11_* for Region i; *W_12_* and *L_12_* for Region ii; *W_13_* and *L_13_* for Region iii; *W_11_* and *L_14_* for Region iv; *W_15_* and *L_15_* for Region v. The height of the channel is notated as *H*.

Based on the Darcy–Weisbach equation and momentum equations for the Hagen–Poiseuille flow, the pressure drop across a length L in a channel can be obtained:

$$\Delta p=fRe\frac{L\bar{u}\eta }{2D_{h}^{2}}.$$

Here *R_e_* is the Reynolds number, *f* is the Darcy friction factor, $\bar{u}$ is the average flow velocity of the fluid, *D_h_* is the hydraulic diameter, *Q* is the volumetric flow rate, *η* is the fluid viscosity. The Reynolds number (*Re*) is defined as:

$$Re=\frac{InertialForces}{ViscousForces}=\frac{\rho \bar{u}D_{h}}{\eta },$$

where *ρ* is the fluid density. The product of the Darcy friction factor (*f*) and the Reynolds number (*Re*) is a constant, which depends on the cross-sectional geometry of the channel [9], [17]. The hydraulic diameter (*D_h_*) is exploited for approximating the flows through these noncircular geometries and is given by:

$$D_{h}=\frac{4A}{P},$$

where *A* is the cross-section area and *P* is the perimeter of the channel that is in direct contact with the flow.

Path 1 contains five regions as indicated in Fig. 1B, therefore, the total pressure drop across Path 1 (from point A to point B) is: *Δp_2_* = *Δp_11_* + *Δp_12_* + *Δp_13_* + *Δp_14_* + *Δp_15_*. The pressure drop in Path 2 is indicated as Δ*p_2_*. The pressure drop between point A and B (in Fig. 1B) is constant, thus, $\Delta p_{1}=\Delta p_{2}$. The volumetric flow rate (*Q*) in a fluid channel is given by: $Q=\bar{u}A$. The design criterion for efficient particle trapping is that the volumetric flow rate along the looped bypass channel (Path 2) should be smaller than that of trapping path (Path 1) [9], i.e., $Q_{2}/Q_{1}<1$. Depending on this criterion, we have derived the dimensions of the channels and traps for the trapping configuration in Fig. 1B. The geometric dimensions are summarised in Table 1. A particular design has a 5 μm trapping gap (design 1), while the other one has a 3 μm trapping gap (design 2). They have different channel dimensions and thus can accommodate particles/cells with different sizes.

Valves are created where a control channel crosses a flow channel. In this work, the width of the trapping channels in the flow layer is 25 μm (design 1) or 15 μm (design 2). The width of the valve channel (control channel) is designed to be 40 μm. Therefore, the effective valve area (i.e., the overlapping region between the middle chamber and the valve channel) is 25 × 40 μm^2^ or 15 × 40 μm^2^. The performance of valves is highly dependent on the cross-sectional profile of the flow channel [11], [18]. If the cross-section of the flow channel is square or rectangular (i.e., with sharp corners), the membrane is not able to completely seal the corners. To create completely sealing valves, flow channels must have a rounded cross-sectional profile at the valve area. To efficiently release trapped particles/cells and preventing re-trapping of other particles/cells, the flow path via the middle chamber of each trapping group (Fig. 1) needs to be blocked completely. Therefore, the channels in the flow layer are chosen to have a rounded cross- section profile so that complete valve sealing of the middle chambers in the channel is possible.

**Fig. 2:**
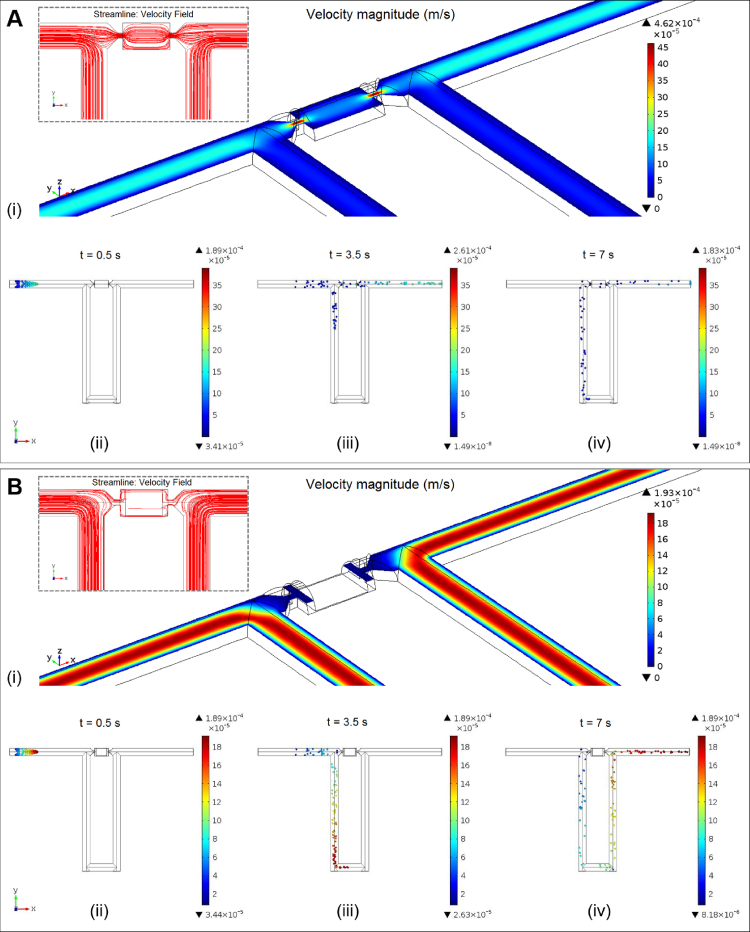
Simulation results showing the flow velocity profiles and particle trajectories in the trapping channels when valve is deactivated (A) and when valve is activated (B). Flow velocity magnitude (m/s) is shown in (i). Insets show the streamlines of the velocity field. Particle trajectories in the trapping channels at different time points after particles being released at the inlet are shown in (ii–iv). Boundary conditions: inlet velocity is set to be 100 μm/s; outlet pressure is set to be 0 Pa; walls are set to the ‘No slip’ wall boundary condition. One hundred particles (diameter = 10 μm; density = 1050 kg/m^3^) are released at the inlet of the channel and driven by the drag force of the fluid. It should be noted that the time-dependent particle tracing simulation is only used to demonstrate the fluid dynamics. The particles released in the simulation are virtual, therefore would not be stopped by the mechanical trap. The particle tracing simulation is used to calculate the probabilities of particles flowing into the trap and into the bypass.

A 3D model has been built and finite element simulations using COMSOL Multiphysics 4.4 have been performed (see Fig. 2) to study the flow velocity profiles in the trapping channels as well as to understand particle trajectories. When the valve is deactivated (i.e., valve off), the flow velocity in the small trapping gap is much higher than the surrounding liquid (Fig. 2A(i)), thereby driving particles into the trap and thus achieving the trapping function. The inset shows the streamlines of the velocity field. The time-dependent particle tracing simulation is shown in Fig. 2A(ii–iv). It should be noted that the simulation is only used to demonstrate the fluid dynamics. The particles released in the simulation are virtual, therefore would not be stopped by the mechanical trap. This is the reason why the particles seem to be injected into Path 1. Nevertheless, this simulation can intuitively tell us the probability of particles flowing into the trap and into the bypass. The criteria for efficient single particle trapping is that the probability of particle flowing into the trap should be larger than that of particle flowing into the bypass. In this case, the transmission probability of particles flowing into the trap is 62%, which is greater than the probability for the particle to flow into the bypass channel, i.e., 38%. This satisfies the criteria for the trap to work efficiently [9]. When the valve function is activated (i.e., valve on) and trapping channel is completely sealed by the valve membrane, there is no flow through the small trapping gap (i.e., flow velocity is zero) and the only flow path existed is through the bypass channel (Fig. 2B(i)). This indicates that when the valve is fully activated, particles would only be driven to the bypass channel by the drag force of the fluid. In this case, the transmission probability of particles flowing into the trap is nearly zero, while the probability for the particle to flow into the bypass channel is 100% (Fig. 2B(ii–iv)).

## 3 Material and methods

### 3.1 Device fabrication

Master moulds for flow layer (containing trapping channels) were fabricated on 3″ silicon wafers using positive resist AZ 9260 (MicroChemicals). After standard photolithography processes, a hard bake was performed at 118 °C for 2 min to reflow the positive resist, thereby rendering the channel cross-section profile to be rounded (Supplementary material Fig. S3). Master moulds for control layer (containing valve channels) were fabricated using negative resist SU-8 2025 (MiroChem). Prior to PDMS (polydimethylsiloxane) moulding, all master wafers were treated with FDTS (1H,1H,2H,2H-Perfluorodecyltrichlorosilane, 96%, Alfa Aesar) by vapour deposition and rendered to be hydrophobic [2]. The FDTS- coated surface is highly hydrophobic, thereby easing the PDMS peeling process from the master mould. Details of master mould fabrications are provided in Supplementary material.

Microfluidic channels were fabricated using multilayer PDMS soft-lithography [11]. A general way of making two-layer PDMS devices is to make a thin PDMS layer on the flow layer mould (by spin coating), and a thick PDMS layer on the control layer mould (by pouring) as a substrate for easy handling. However, it has been shown that thick-layer PDMS structures will shrink when the thick PDMS layer is peeled away from the master wafer. Shrinkage is mainly due to the thermal expansion and contraction during the curing process where elevated temperature is usually used to reduce the curing time required. The shrinkage of thick-layer features causes misalignment of different PDMS layers. One solution to the shrinkage problem is to cure the PDMS at room temperature. However, it takes several days for the PDMS to be fully cured at room temperature and achieve full mechanical strength. This significantly reduces the throughput for device fabrication. Besides, the PDMS shrinkage problem may still occur as the temperature varies. Another possible solution is to scale up the mask for the thick layer (generally the control layer). However, the shrinkage of PDMS is dependent on many factors, such as the composition, baking temperature and time and may differ from batch to batch. Especially for applications where high alignment accuracy is required, simply scaling up the mask by a fixed factor may not lead to satisfactory and repeatable results every time.

In this work, instead of making thin and thick PDMS layers, we spin-coated PDMS on both control and flow master wafers, resulting in two thin PDMS layers. These PDMS layers are thin enough that shrinkage can be ignored even when the PDMS is cured at elevated temperature (up to 150 °C as recommended by the manufacturer). The thin PDMS layers were bonded to a thick PDMS substrate for easy handling, finally bonded to a glass substrate to close the channels. We find that this approach can efficiently solve the shrinkage-induced PDMS registration problem for multilayer PDMS fabrication. Sylgard 184 silicone elastomer kit (Dow Corning) was used for multi-layer PDMS soft-lithography in this work. The detailed multi-layer PDMS fabrication process is summarised in Supplementary material and schematically shown in Fig. S2.

### 3.2 Experiment setup

#### 3.2.1 Particle trapping and release

The fabricated device was connected to a 1 ml syringe with polytetrafluoroethylene (PTFE) tubing (0.59 mm ID × 0.25 mm Wall) and 23 G needles. The flow was controlled by syringe pumps. Prior to use, the microfluidic device was flushed in PBS (phosphate-buffered saline) with 10% Tween 20 for an hour. Incubating PDMS surface with Tween 20 could effectively avoid hydrophobic interactions between polystyrene (PS) particles and the channel surface. Polystyrene particles with different sizes (8 μm, 10 μm, 12 μm, 15 μm) were prepared and diluted in 0.05% Tween 20 in PBS, resulting in a concentration of 36 particles/μl. The prepared particle solutions were sonicated to avoid particle aggregation. After the PDMS surface was treated with Tween 20, polystyrene particles were injected into the device at a flow rate of 20 μl/h. The trapping process was observed from a microscope. After all traps were filled with particles, the device was washed with PBS or DI water. Particle release was done by activating the valve channels in the control layer: DI water was infused into the control channel until a sufficient pressure was built up to fully deflect the valve membrane. Syringe pumps were used in this work, therefore, the valve was activated by controlling the flow rates rather than the pressure. The flow rate in the control layer was kept at more than 600 μl/h to pressurise the channel, causing valve membrane to deflect. The flow rate in the trapping channels was kept at a much lower value (e.g., 10 μl/h) to minimise the back pressure built up inside the flow channel.

#### 3.2.2 Cell trapping and release

Mouse embryonic stem cells (mESCs) or their nuclei were prepared as described in Supplementary material. Before use, the microfluidic device was pre-treated with 1% BSA (bovine serum albumin) in 1 × PBS for 30 min to block hydrophobic interactions between the cells and PDMS surfaces. After the treatment, cells were driven into the device at a flow rate of 20 μl/h by a syringe pump. The trapping process was monitored with microscopes. After cells were trapped, the flow channel was washed with PBS buffer at a flow rate of 20 μl/h. Cell release was carried out by pressuring the valve channels with DI water, similar to the procedures for releasing particles.

#### 3.2.3 Imaging of mEos3-tagged Cenpa and tdiRFP- tagged histone H2B

Imaging was carried out using on a custom built super-resolution imaging setup similar to ones published previously [2]; the collimated and circularly polarized output of a 641 nm (Cube 640-100C, Coherent), 561 nm (Cobolt, Jive 200), 488 nm (Toptica, iBeam Smart 488 100 mW), and 405 nm (Oxxius, LaserBoxx 405) laser beams that were aligned and focussed at the back aperture of an Olympus 1.49 NA 60x oil objective mounted on an IX71 Olympus inverted microscope frame. The fluorescent signal was filtered with a four-band dichroic (Semrock, Di01-R405/488/561/635) and then the relevant emission filter (described below) before being expanded through a 2.5× achromatic beam expander (Olympus, PE 2.5 × 125) and finally projected onto an EMCCD (Photometrics, Evolve 512). 3D imaging of Cenpa and histone H2B were carried out by taking z-stacks separated by 100 nm over a 14 μm range using a piezo objective positioner (C-focus, Mad City Labs). The green form of mEos3-tagged Cenpa was imaged using 488 nm excitation (power density of 1 kW cm^−2^) and the emission filtered using a combination of a 488 long-pass filter (Semrock, BLP01-488R) and a band-pass filter (Semrock, FF01-520/35). tdiRFP-tagged H2B was imaged using 641 nm excitation (power density of 100 W cm^−2^) and the emission filtered using a 641 long-pass filter (Semrock, BLP01-635R). The plane at which the centromeres were positioned in the z direction was determined by processing of these 3D stacks using custom-written software. The images of the z-stack were normalized to have the same mean background intensity (i.e., intensity outside a rectangular region of interest containing the cell). To increase the contrast of points, especially along the *z*-axis, deconvolution was performed using the SALSA algorithm [19]. For point features such as centromeres, a sparse (l1) regulariser was used, and for diffuse features (e.g., histone H2B) a total-variation regulariser was used. Both were implemented as suggested in the original paper. To colour the images by depth, a colour was defined for each layer from a terrain colour map (colours varying smoothly from blue to green to yellow to brown). The entire layer was then multiplied by that colour, and a sum along the *z*-axis was taken to generate a 2D image. If the resulting spots were too small to be easily visible, a Gaussian blur with a 1-pixel radius was applied using the GNU Image Manipulation Program.

2D super-resolution imaging of centromeres was carried out on cells expressing mEos3-tagged Cenpa by PALM imaging of the red photoactivatable form of mEos3 as previously described [20]. Briefly, 5000 frames were taken at 50 ms exposure using 561 nm and 405 nm excitation lasers (power densities of 10 W cm^−2^ and 10–100 W cm^−2^ respectively), and the emission filtered using a combination of a 561 band-pass (Semrock, BLP01-561R) and a band-pass filter (Semrock, FF01-587/35). Data was analysed using Rapidstorm [21].

## 4 Results and discussion

### 4.1 Particle trapping and release

Due to the geometric symmetry of the device, particles can be captured either at the right trap or at the left trap, depending on the flow direction (Fig. S8 in Supplementary material). The trapping of single particles is highly efficient and repeatable (Fig. S4 in Supplementary material). It is found that the single-particle trapping efficiency (the ratio of the number of traps that are occupied by single particles to the total number of the traps in the device) can reach more than 95% with optimised design. Furthermore, the trapping efficiency is not influenced by the flow rate used in the experiment. Therefore, a wide range of flow rates could be used in this device to achieve efficient single-particle trapping. In addition, particles could be trapped in the device for a long period of time and would not escape from the traps.

We tested devices with different channel dimensions and studied the trapping efficiency for particles with different sizes, ranging from 8 μm to 15 μm in diameter. For trapping efficiency measurements with different devices, the valve layer is not included in order to simplify the fabrication process (it has been observed that the trapping efficiency of the device with the valve layer does not differ much from the efficiency measured from the device without the valve layer, indicating that the integration of valve layers does not influence the trapping efficiency of the device).

**Fig. 3:**
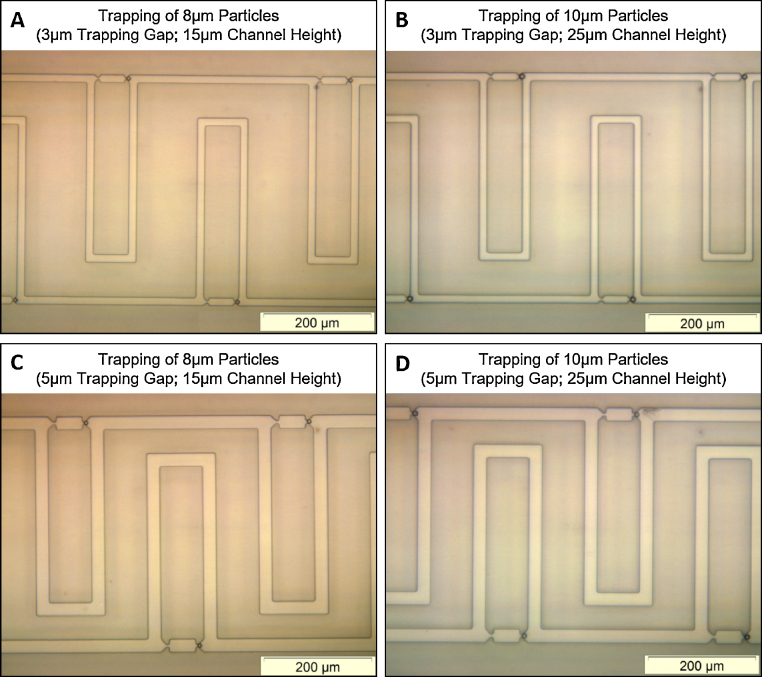
Particle trapping in devices with different channel dimensions. (A) Trapping of 8 μm particles using the device with 3 μm trapping gap and 15 μm channel height. (B) Trapping of 10 μm particles using the device with 3 μm trapping gap and 25 μm channel height. (C) Trapping of 8 μm particles using the device with 5 μm trapping gap and 15 μm channel height. (D) Trapping of 10 μm particles using the device with 5 μm trapping gap and 25 μm channel height.

Examples of polystyrene particle trapping are illustrated in Fig. 3, where devices with different channel dimensions were tested and particles with different sizes were used. Some of the results are summarised below: (**i**) trapping of 8 μm particle using the device with 3 μm trapping gap and 15 μm channel height (Fig. 3A): almost all of the traps (>95%) could achieve single particle trapping; (**ii**) trapping of 10 μm particle using the device with 3 μm trapping gap and 25 μm channel height (Fig. 3B): most of the traps (∼90%) could achieve single-particle trapping, and ∼10% traps captured two particles. The reason is that the channel height (25 μm) is about twice of the particle size (10 μm). Therefore, two particles (10 μm) can be accommodated into the same trap (in the vertical direction, and thus cannot be seen from the figure); (**iii**) trapping of 8 μm particle using the device with 5 μm trapping gap and 15 μm channel height (Fig. 3C): approximately 90% of the traps could enable single particle trapping; a few traps immobilise two particles (not shown in the figure) which may be explainable by the fact that the width of the channel at the entrance of the trap (25 μm) is more than twice of the particle diameter (10 μm); (**iv**) trapping of 10 μm particle using the device with 5 μm trapping gap and 25 μm channel height (Fig. 3D): approximate 80% of the traps could achieve single particle trapping, while the other ∼20% traps captured two particles (or even three particles), though not shown in the figure. The reason behind is that both the channel height (25 μm) and the channel width (25 μm) are about twice of the particle size (10 μm). Therefore, two or three 10 μm particles can be accommodated into the same trap (either in vertical direction or in horizontal plane).

It is observed that the efficiency of single particle trapping is dependent on both the channel dimensions and the size of the target particles. Therefore, it is important to optimise the channel dimensions, in order to achieve the best and most efficient trapping of particles and cells that are of interest.

For particles smaller than 12 μm, it is found that the design with the 3 μm trapping gap is more efficient than the one with 5 μm trapping gap in terms of single-particle trapping (for particles smaller than 12 μm). However, due to the narrow channels, the 3 μm gap design suffers higher risk of channel blockage. The main and bypass channels in the 5 μm-gap device are 25 μm wide, larger than twice the particle size, thereby increasing the probability of immobilising multiple particles in one trap. For particles larger than 12 μm, the blockage problem in the 3 μm-gap device make the particle trapping difficult or even impossible. On the other hand, the 5 μm-gap device demonstrates good performance in trapping relatively large particles (12–20 μm in diameter).

We also studied the influence of channel height on the trapping efficiency of single particles. We fabricated and tested devices with channel height of 15 μm and 25 μm (for both the 3 μm-gap design and 5 μm-gap design). For particles smaller than 12 μm, the devices with 15 μm channel height gives better results in terms of single-particle trapping. The devices (both 3 μm gap and 5 μm gap) with 25 μm channel height, which is larger than twice of the particle size, usually results in multiple particles immobilised in the same trap (examples are shown in Supplementary material Fig. S5). However, these devices (25 μm channel height) perform well when trapping relatively large particles (12–20 μm in diameter).

In summary, both the trapping gap dimension and channel dimensions can influence the efficiency of particle trapping. The trapping gap should always be smaller than the size of target particles in order to physically immobilise a particle and prevent it from flowing through the gap. Smaller gap usually results in higher trapping efficiency. However, the trapping efficiency also depends on the lengths of the straight channel and bypass channel (based on the flow resistance theory). The width and height of the channel are also important in determining the trapping results. To achieve highest efficiency for single-particle trapping, the channel width and height should be larger than the particle size, but smaller than twice of the particle size to avoid multiple- particles being trapped in the same trap. However, decreasing channel dimension may increase the chance of channel blockage. Using diluted particle samples and lowering the flow rate may help reducing the chance of blockage. Sonicating particles before trapping (reducing particle aggregation) can also help. It is also found from the experiments that the channel height seems to play a more important role in determining the single-particle trapping efficiency than the channel width. In other words, decreasing the channel height may have a more pronounced effect in increasing the trapping efficiency than decreasing the channel width. Therefore, to avoid channel blockage, the channel width can be kept relatively large, whereas the channel height should be kept slightly larger than the target particle size but smaller than twice the particle size.

Particle release was successfully demonstrated by pressuring the valve channels in the control layer and activating the valve function. Control channels are filled with DI water instead of air to prevent air from diffusing through the valve membrane and introducing bubbles into the flow layer. Due to the gas permeable properties of PDMS, the dead-end control channels will be depleted of air as the fluid is pushed into the control channels, leading to a bubble-free fluid-filled control channel. Once the control channels are filled, the microfluidic valves can fully close the channels in the flow layer if a further pressure is applied to overcome the PDMS elasticity and the back pressure in flow layer. Valve sealing can be observed at the overlapping region for the valve channel and trapping channel (Fig. S6). When the pressure in the control channel is relieved, the elasticity of PDMS causes the valve membrane to spring back to its original position, deactivating the valve function and opening the channels in flow layer. Before trapping particles, the valve function is tested with DI water, and it is found that all the valves fabricated using the proposed protocol performed very well (see Figs. S6 and S7 in Supplementary material).

**Fig. 4:**
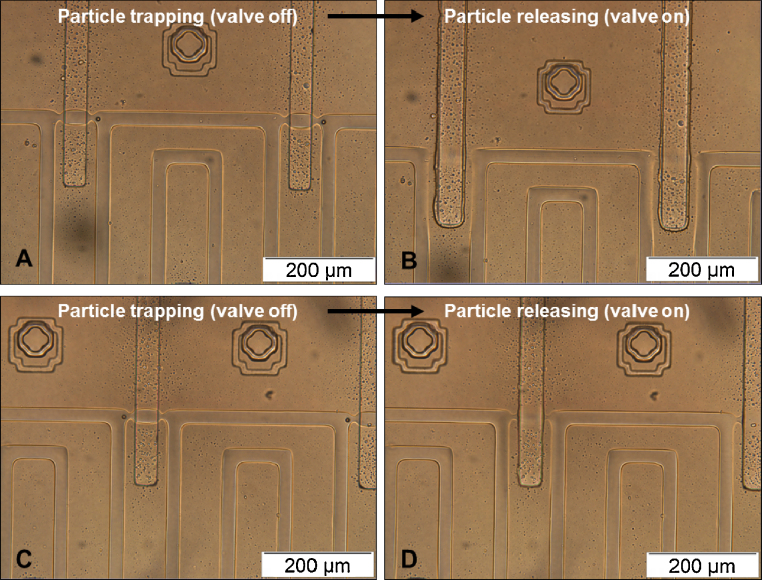
Examples of particle trapping and release. Images in the left column (A,C) show the trapping of single particles when valves are deactivated. The release process of these particles can be done by activating corresponding valves and sealing corresponding middle chambers in the flow layer. Images in the right column (B,D) show the results of particle released from (A,C), respectively. All particles are found to be released from the traps when the valves are activated and the flow paths through middle chambers are closed. Due to the geometric symmetry of the device, particles can be captured either at the right trap or at the left trap, depending on the flow direction. (A) and (B) show the examples when flow direction is from right to left, while (C) and (D) show the situation when flow is from left to right.

After verifying that the valves function well, particle trap and release experiments are performed. Polystyrene particles are successfully trapped and released from the trapping site by controlling the valves. Fig. 4 shows examples of single particle trap and release. Images in the left column (A,C) show the normal trapping of single particles when valves are deactivated (i.e., valve off). Images in the right column (B,D) demonstrate that particle release can be done by pressurising the valve channels in the control layer and sealing the middle chambers in the flow layer (i.e., valve on). All particles are found to be successfully released from the traps when the valves completely seal the middle chambers. Time-lapse images of the particle release process were recorded (Fig. S9 in Supplementary material), demonstrating that particles can be released and the sequence of the particles being released does not influence the release process. In Tan’s work [9], a predetermined order (from upstream to the downstream of the flow) is necessary for the particles to be released successfully. Failure to follow this order would result in released particles being re-trapped in empty traps in the downstream. On the contrary, the design presented in this work allows particles to be released with any order or at any time by controlling the valve states (i.e., on or off). This enables single particle trapping, release and manipulation in a controllable manner. Moreover, the particle release process is not (or less) dependent on the particle properties (e.g., diameter or mass) and fluid properties. Once the valve is fully activated, there is only one possible path for the particle to flow in the channel. Particles will be driven out of the trap by the drag force of the fluid, which can be controlled by the flow rate.

We also tested the particle trapping performance in the device when valves are activated. No particle was trapped when the valves completely sealed the middle chambers of each trapping unit in the flow layer. When valves are activated, all particles flow past the trap (Fig. S10 in Supplementary material) through the bypass channel. This means that particle/cell trapping can also be controlled by the valve. Particles can be trapped at particular traps and positions by setting the corresponding valves to be off (i.e., relieving the pressure in the control layer and opening the middle chamber in the flow layer). On the other hand, particles could be forced to bypass a trap and not be trapped if the corresponding valve is activated (i.e., pressurising the valve channel in the control layer and sealing the middle chamber in the flow layer). Similarly, particles can be selectively released by controlling the valves. This feature can be beneficial to many applications. For example, by simply controlling the valves, different types of particles/cells can be trapped in desired positions, and more importantly in the same device. This would enable simultaneous analysis of different samples in the same device. Another application is for single cell analysis. Single cells can be first trapped at desired positions for preliminary analysis, such as high-resolution imaging or electrical impedance analysis, which requires cells being fixed at a position and not moving during the whole imaging process. After the analysis step, cells can then be released by simply activating corresponding valves, and driven into the next stage of the device for further culturing or other processes. More importantly, this design enables selectively releasing cells that are of interest, without affecting other cells being trapped.

### 4.2 Cell trapping and release

The diameter of stem cells and their nuclei can range from 8 μm to 12 μm. Both the 3 μm-gap design and 5 μm-gap design can be used to trap these cells. Based on the particle trapping experiments, the 3 μm-gap design offers a higher single- particle trapping efficiency. However, as mentioned before, the 3 μm-gap design also suffers from a higher risk of channel blockage compare with the 5 μm-gap design (because the main channel of the 3 μm-gap design is narrower than that of the 5 μm-gap design). To minimise channel blockage, the 5 μm-gap device (with a channel height of 15 μm) was chosen for most of the cell trapping experiments.

**Fig. 5:**
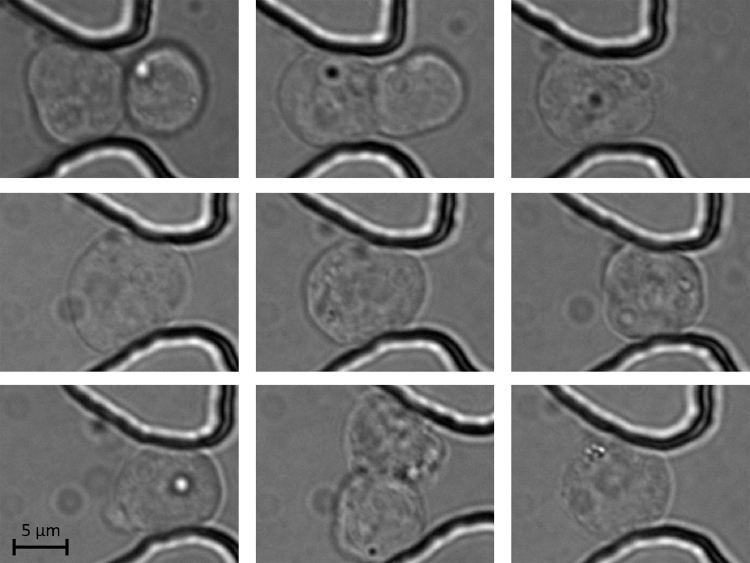
Mouse embryonic stem cell nuclei are immobilised at different trapping locations inside the device. The trapped cell nuclei can be kept in place up to several days without leaving their original trapping positions. Scale bar: 5 μm (for all the sub-panels).

**Fig. 6:**
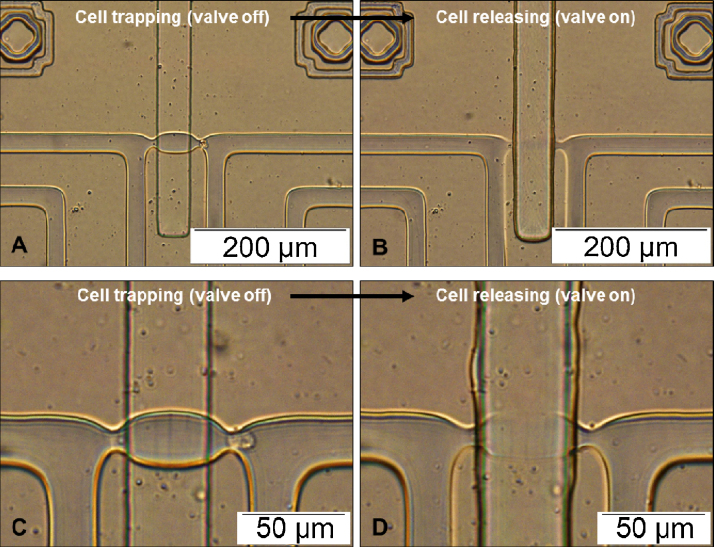
Trap and release of mouse embryonic stem cells (mESCs). Flow direction in the trapping channels is from right to left. Images in the left column (A,C) show the trapping of single cells when valves are deactivated. Cell release can be done by activating corresponding valves and thus stopping the flow paths through the middle chambers. Images in the right column (B) and (D) show the results of cell released from (A) and (C), respectively.

We have demonstrated experimentally that mESCs or mESC nuclei can be efficiently trapped in the device (Fig. 5) and kept in place up to several days without leaving their original trapping positions. Cell release can be done by using the same method as that for releasing polystyrene particles. Examples of cell trapping and release are shown in Fig. 6. Flow direction in the trapping channels is from right to left. Images in the left column (A,C) show the trapping of single cells when valves are deactivated. Cells are released from the trap by activating corresponding valves and thus stopping the flow paths through the middle chambers. Images in the right column (B) and (D) show the results of cell released from (A) and (C), respectively. We have found that the valves are intact after the release process. Therefore, the trap and release process can be repeated and the device can be reused.

Compared with Tan’s method of releasing particles [9], [10] (where aluminium electrodes are deposited underneath a chamber closed to the trap and a laser is used to generated heat and form bubbles inside the chamber to push out the particle from the trap), the method proposed in this work significantly eases the fabrication process and simplifies the experiment setup required for releasing particles and cells. Additionally, this method does not generate heat and will therefore have minimal impact on biological samples like cells. Moreover, in this design, particles or cells can be selectively released from the traps in any order and at any time by simply activating the corresponding valve. If there is an empty trap in the path the released particle flows through, the particle/cell can be prevented from being re-trapped by simply activating the corresponding valve of the empty trap (close the middle chamber of the trapping channel in the flow layer and thus stop the flow in that path). Similarly, if the particles or cells are required to be trapped at a particular location, this could be done by deactivating the valve function of the corresponding trap. In summary, particles or cells can be trapped at any location and released at any time or moved to other traps by controlling the corresponding valves.

### 4.3 Super-resolution imaging

**Fig. 7:**
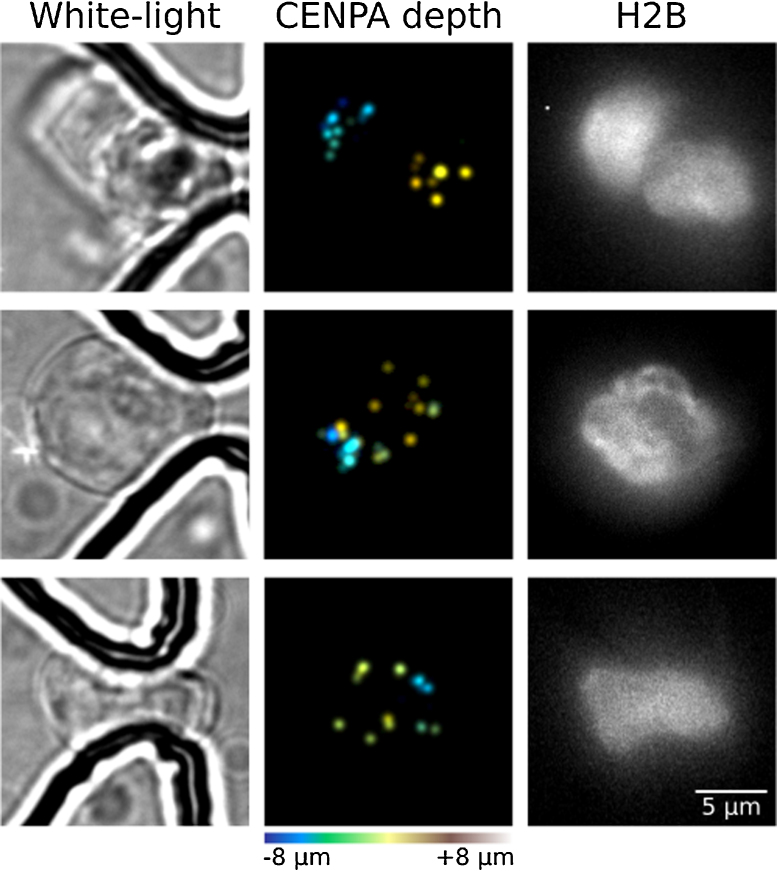
Trapping and conventional 3D imaging of single mouse embryonic stem cell (mESC) nuclei. White light and iRFP-tagged histone H2B images of the trapped nuclei are shown left and right respectively. The centromeres (imaged using mEos3-tagged Cenpa) are shown in the middle with each centromere colour-coded to represent its position in the *z*-axis after running the 3D stacks through custom-written deconvolution software.

**Fig. 8:**
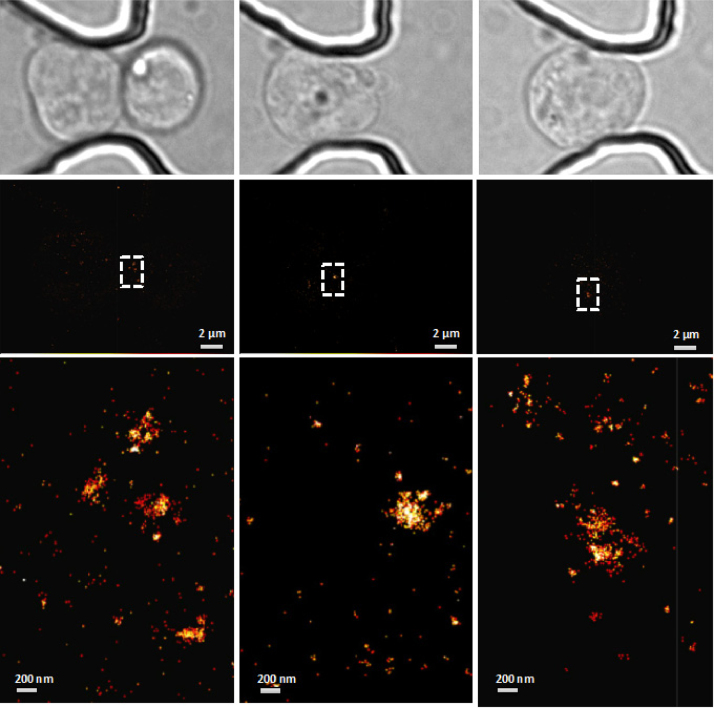
2D super-resolution imaging of mEos3-tagged Cenpa in haploid mouse embryonic stem cell nuclei. Nuclei were trapped in the device and imaged as described. The white-light image of the trapped cell is shown above with the reconstructed super-resolution image shown in the middle. By zooming into the indicated white dotted square, it is possible to identify centromeres of ∼200 nm size with a localisation precision of <15 nm as shown in the zoomed images below which has been colour-coded by density from low to high being represented from red to yellow to white. (For interpretation of the references to colour in this figure legend, the reader is referred to the web version of this article.)

Super-resolution imaging of single mESCs and nuclei have been performed successfully. Fig. 7 shows the trapping and conventional diffraction-limited 3D imaging of haploid mouse embryonic stem cells expressing mEos3-tagged Cenpa (to label centromeres) and iRFP-tagged histone H2B (to label the nuclei) in a microfluidic trapping device. Centromeres in some cells were then placed in the focal plane and imaged using super-resolution PALM microscopy. The positions of single Cenpa protein molecules were determined to have a localisation precision of 12.3 (±1.0) nm using nearest neighbour analysis software previously described [22]. This precision value is comparable to that observed in previous 2D super-resolution imaging studies of mEos3 [20], [23] and to mEos3-tagged Cenpa imaged in mESCs sandwiched between coverslips when grown on 0.1% gelatin (Fig. S11 in Supplementary material). Fig. 8 shows the 2D super-resolution imaging of mEos3-tagged Cenpa in haploid mouse embryonic stem cell nuclei. Nuclei were trapped in the device and imaged as described. The white-light image of the trapped cell is shown above with the reconstructed super-resolution image shown in the middle. By zooming into the indicated white dotted square, it is possible to identify centromeres of ∼200 nm size, as shown in the bottom row. The microfluidic cell trapping technique described in this work has the benefit that a large number of cells can be trapped and imaged at the same time, therefore saving a lot of time and effort when compared to the traditional way of picking cells where each cell has to be individually picked manually using micropipettes. This trapping method thus significantly eases the experiment setup for single-cell characterisation and increases the throughput for such studies.

## 5 Conclusions

A multi-layer microfluidic device allowing trapping and release of single particles/cells in a controllable manner is demonstrated. This device combines hydrodynamic trapping with microfluidic valving techniques. The proposed design has the advantage that single particles/cells can be trapped, released and manipulated by simply controlling corresponding valves. Particles and cells could be trapped normally when the valve function is not activated. After being trapped and analysed, particles/cells can be selectively released by activating corresponding control valves. The controllable trap and release feature of this device can be beneficial for many applications. For example, by simply controlling the valves, different types of particles/cells can be trapped in desired positions and simultaneous analysis of different samples in the same device is possible. Similarly, by controlling the valve, this design enables selectively releasing the particles/cells that are of interest, without affecting other samples being trapped. Thus, this feature potentially allows for data from super-resolution imaging to be integrated with other studies to be conducted on the same cell, either on the same microfluidic substrate or elsewhere. The trap and release processes are quite reliable and repeatable, and more importantly, do not result in any harmful effects on biological samples such as cells. Cells were successfully trapped and held in place over several days. Real-time and long-term monitoring of individual mESCs and mESC nuclei using super-resolution imaging has also been demonstrated. It is possible to identify centromeres of ∼200 nm size with a precision of <15 nm. The proposed trapping device has great potentials in single cell analysis, such as super-resolution imaging, where cells are required to be precisely immobilised at a position over long periods of time.

## Figures and Tables

**Table 1:** Geometric channel dimensions (unit: μm).

	*L*_11_ (*L*_14_)	*L*_12_ (*L*_15_)	*W*_12_	*W*_13_	*L*_13_	*W*_2_	*L*_2_	*H*
Design 1	10	5	5	25	50	25	805	15 or 25
Design 2	6	3	3	15	48	15	761	15 or 25
