# Supplementary material for: A microfluidic platform for trapping, releasing and super-resolution imaging of single cells
Source: Sens Actuators B Chem. 2016 Sep;232:680–91. doi: 10.1016/j.snb.2016.03.131 (PMC4872524; doi:10.1016/j.snb.2016.03.131)
Supplement: Supplementary file 1 [file mmc1.docx]

## Appendix A. Supplementary data

Supplementary material is available.

# Supplementary Material

## Valve Design Overview


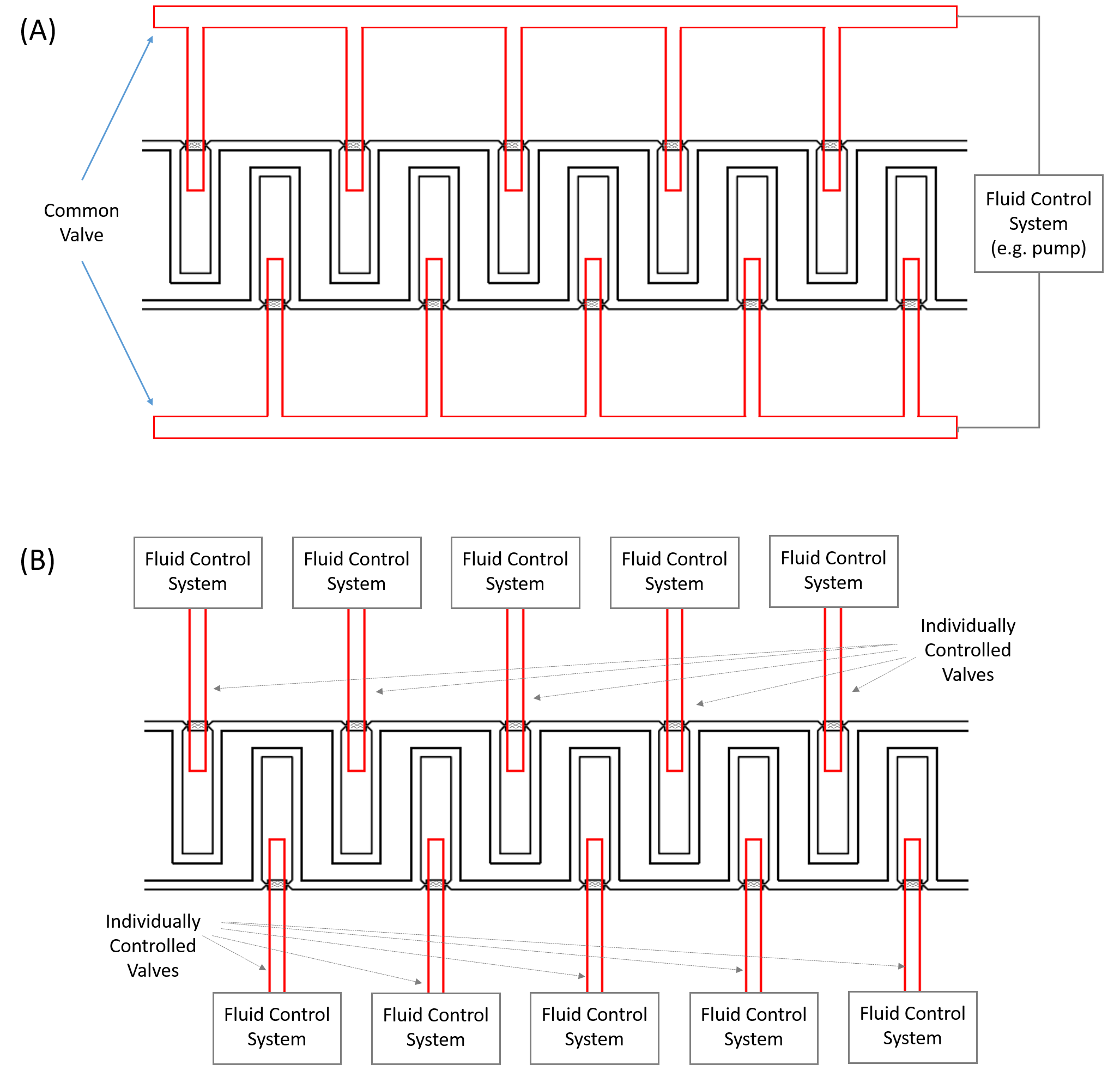


Fig. S1: Overview of the valve designs. (A) All the traps are controlled by a common valve. High throughput particle/cell trapping and release could be done by simply controlling the common valve. (B) Individually controlled valves. In this case, each valve has separate inlet and can be controlled individually by fluid control systems. Selectively trapping and releasing the particles/cells can be possible by activating or deactivating the corresponding valve located on top of each middle chamber of the trapping site.

## Device Fabrication

Fabrication process can be summarised as: i) master wafer fabrication for flow layer (rounded channel profile); ii) master wafer fabrication for control layer (rectangular channel profile); iii) PDMS moulding for flow and control layers; iv) multi-layer PDMS alignment and bonding.

*(1) Master Wafer for Flow Layer (Trapping Channels)*

Rounded profiles are formed using the positive resist AZ 9260 (MicroChemicals), which can be melted and reflowed by hard baking above a certain temperature, causing the initial rectangular profile to become rounded (parabolic). AZ 9260 photoresist was spin coated on 3” silicon wafers at 500 rpm for 8s, then ramp up to 1500 rpm at 1000 rpm/s and hold for 60, resulting in a 12 µm-thick resist film. Thicker film (e.g. 25 µm-thick film) could done by reducing the spin speed or double coating the resist. The coated wafer was levelled and left for stress relaxation for 2 minutes before soft baked on a 110°C hotplate. After soft bake, the resist film was relaxed for 8 hours at room temperature to rehydrate. UV exposure was performed with Karl Suss MJB4 mask aligner at an exposure dose of 800 mJ/cm^2^. After exposure, the resist was developed in AZ 726MIF developer, rinsed with DI water and dried with nitrogen. Finally, a hard bake at 118°C for 2 minutes was performed to reflow the resist and thus render the channel cross-section profile to be rounded (Fig. S3). The reflow bake will increase the height of the photoresist an average of 2~4 μm, depending on the dimensions of the patterned structures.

*(2) Master Wafer for Control Layer (Valve Channels)*

SU-8 2025 (MiroChem) was used for fabricating the control layer master wafer. SU-8 2025 photoresist was spin-coated onto 3” silicon wafers at 500 rpm for 8 seconds and 3000 rpm for 60 seconds, resulting in a 25 µm-thick resist film. Soft bake was performed at 65°C for 1 minute and 95°C for 5 minutes. Exposure was done with Karl Suss MJB4 mask aligner (exposure energy: 150 mJ/cm^2^). After exposure, post bake was performed at 65°C for 1 minute, 95°C for 4 minutes. The wafer was then developed in SU-8 developer (PGMEA) for 4 minutes (with agitation), rinsed thoroughly with propanol and dried with nitrogen. A hard bake step was performed at 200°C for 5 minutes to improve the mechanical properties and thermal performance of SU-8.

*(3) Multilayer PDMS Fabrication*

A general way of making two-layer PDMS devices is to make a thin PDMS layer on the flow layer mould (by spin coating), and a thick PDMS layer on the control layer mould (by pouring) as a substrate for easy handling. However, it has been shown that thick-layer PDMS structures will shrink when the thick PDMS layer is peeled away from the master wafer. Shrinkage is mainly due to the thermal expansion and contraction temperature during the curing process where elevated temperature is usually used to reduce the curing time required. The shrinkage of thick-layer features causes misalignment of different PDMS layers. One solution to the shrinkage problem is to cure the PDMS at room temperature. However, it takes several days for the PDMS to be fully cured at room temperature and achieve full mechanical strength. This significant reduce the turnout of device fabrication. Besides, the PDMS shrinkage problem may still occur as the temperature varies. Another possible solution is to scale up the mask for the thick layer (generally the control layer). However, the shrinkage of PDMS is dependent on many factors, such as the composition, baking temperature and time. Therefore, the shrinkage may differ from batch to batch. Especially for applications where high alignment accuracy is required, simply scaling up the mask by a fixed factor may not lead to satisfactory and repeatable results every time. In this work, instead of making a thin and a thick PDMS layers, we spin-coated PDMS on both control and flow master wafers, resulting in two thin PDMS layers. These PDMS layers are thin enough that shrinkage can be ignored even when the PDMS is cured at elevated temperature (up to 150 °C as recommended by the manufacturer). The thin PDMS layers were bonded to a thick PDMS substrate for easy handling, finally bonded to a glass substrate to close the channels. We find that this approach can efficiently solve the [shrinkage-induced PDMS registration problem](http://www.rsc.org/binaries/LOC/2008/PDFs/Papers/065_0918.pdf) for multilayer PDMS fabrication. The detailed multi-layer PDMS fabrication process is summarised below and schematically shown in Fig. S2.

Prior to PDMS moulding, all master wafers were treated with FDTS (1H,1H,2H,2H-Perfluorodecyltrichlorosilane, 96%, Alfa Aesar) by vapour deposition and rendered to be hydrophobic [1]. The FDTS-coated surface is highly hydrophobic, thereby easing the PDMS peeling process from the master mould. Sylgard 184 silicone elastomer kit (Dow Corning) was used for soft-lithography. The control layer (containing valve channels) was made by mixing 5:1 base and curing agent, and the flow layer (containing trapping channels) was made by mixing 30:1 base and curing agent. Both mixers were first degassed for ~ 60 mins before further process. For control layer fabrication, the degassed PDMS mixture (5:1 base to curing agent ratio) were spun onto the control layer master wafer at 500 rpm for 8 seconds and then 800 rpm for 60 s (Fig. S2A). This layer was cured on a 65°C hot plate for 2 minutes and on a 150°C hot plate for 5 minutes. A separate thick PDMS substrate was made by mixing base and curing agent with 10: 1 ratio, degassing the mixture for 1 hour and then pouring the mixture onto a FDTS-coated blank wafer. After curing, the thick 10:1 PDMS substrate was peeled-off from the blank wafer (Fig. S2B), and bonded to the 5:1 PDMS layer immediately after oxygen plasma treatment (Fig. S2C). The bonded sample was baked in a 65°C oven for 30 minutes to improve the bonding strength, after which the PDMS was peeled off from the control layer mould (Fig. S2D).

For flow layer fabrication, PDMS (30:1 base to curing agent ratio) were spun onto the flow layer master wafer at 500 rpm for 8 seconds and then 2500 rpm for 60s, resulting in a thin PDMS layer of 24 um thickness (Fig. S2E). This layer was cured on an 80°C hot plate for 40 minutes. Since the structures on the flow layer master wafer are 14 ~ 16 um in height, spinning 24 um PDMS results in a valve membrane of 8 ~ 10 um thickness at the overlapped area between the trapping channel and valve channel. The membrane thickness is chosen so that the membrane is thick and stable enough for fully separating the channels from the control layer, but still thin enough to allow the flow channels to be pinched shut under positive pressure in the control channel above.

The alignment and bonding of the control layer and flow layer was done using a mask aligner. Before bonding, the surfaces of PDMS samples were activated by oxygen plasma. The thick PDMS sample containing valve channels was attached to a custom-made acrylic holder, which was held in position by the vacuum mask holder of the mask aligner. The 30:1 thin PDMS layer was still on the flow layer master wafer. The wafer was loaded into the standard wafer chuck of the mask aligner. The alignment process is similar to the ordinary process of aligning photo-masks and wafers during photolithography. Once the alignment of the flow layer and control layer was done, the two PDMS pieces were brought into conformal contact to form permanent bonding (Fig. S2F). The whole alignment and bonding process was done within 15 minutes after the plasma activation. The alignment errors using this method were typically smaller than 5 µm. After the alignment and bonding, the sample was cured on an 80°C hot plate for 30 minutes to strengthen the bonding between PDMS layers. After baking, the PDMS sample was then peeled off from the flow layer master wafer and cut into desired shape (Fig. S2G). Inlet and outlet holes were drilled by a biopsy punch. Finally, the PDMS sample and a glass substrate was activated by oxygen plasma (Diener etcher) and bonded together permanently to close the channels in the flow layer (Fig. S2H). The final device was again cured in a 65°C oven for 1 hour to improve bonding strength between PDMS and glass.


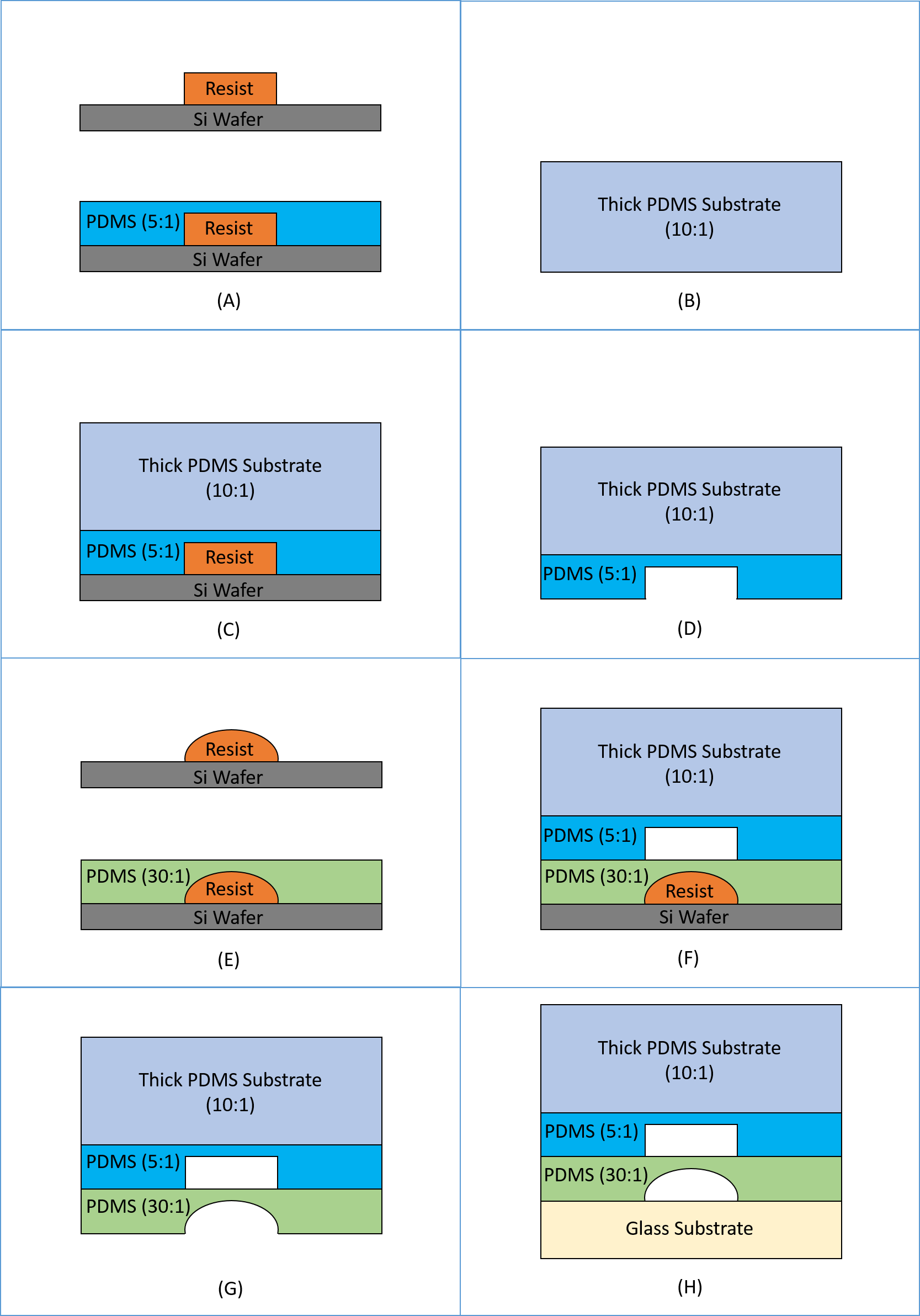


Fig. S2: Schematic diagram showing the fabrication process of the multi-layer PDMS device. (A) PDMS (5:1) is spin coated onto the control layer master wafer (containing valve channels) and cured. (B) A thick PDMS (10:1) substrate is made and serve as a base for thin PDMS layers for easy handling. (C) The control layer is bonded to the thick PDMS substrate immediately after oxygen plasma activation of the bonding surfaces. (D) Release the bonded PDMS sample from the control channel mold. (E) Spin coating PDMS (30:1) onto the flow layer master wafer (containing trapping channels) and half cured at 80°C for 40 minutes. (F) After oxygen plasma activation, the control layer and flow layer are aligned and bonded together. Bonding strength can be improved by post baking the sample at 80°C for half an hour. (G) The PDMS sample is released from the flow layer master wafer. Holes are drilled to form inlets and outlets for fluid. (H) The PDMS sample is finally bonded to a glass substrate to seal the channels in the flow layer.


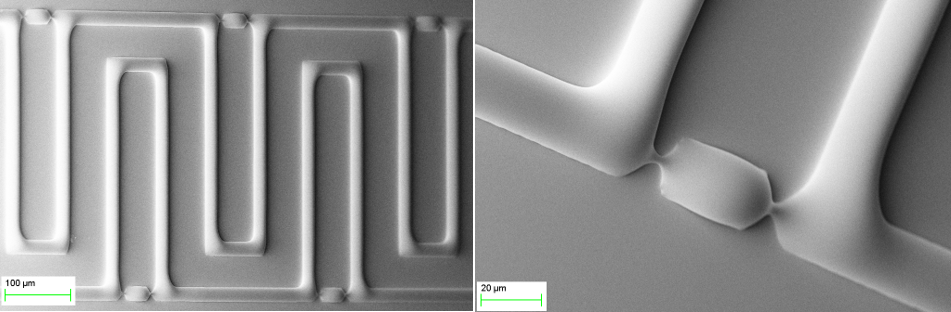


Fig. S3: SEM images of the flow layer master wafer after hard bake at 118°C for 2 minutes. The trapping channels formed by the AZ 9260 have rounded cross-section profiles, due to the reflow of positive resist at high temperature.

## Cell culture and cell line generation

Haploid mouse embryonic stem cells (mESCs) were kindly donated by Martin Leeb (Anton Wutz, Austin Smith). They were cultured on 0.2 % gelatin in 2i media (NDiff B27 base medium, Stem Cell Sciences Ltd, cat. SCS-SF- NB-02, supplemented with 1 μM PD0325901, 3 μM CHIR99021 and 20ng/ml LIF) and sorted every 4 passages to enrich for haploid cells as previously described [2]. Haploid mESCs expressing mEos3.2-tagged Cenpa and iRFP-tagged histone H2B were generated as follows. The mammalian vector expressing mEos3-tagged Cenpa has been previously described [3] and the vector expressing tandem iRFP tagged histone H2B was obtained from Addgene (Maria-Elena Torres-Padilla; Addgene plasmid 47884) [4]. Cell lines were generated by transfecting the mEos3-Cenpa plasmid or tdiRFP-H2B plasmid using lipofectamine 2000 followed by selection in 500 µg/ml geneticin for mEos3-Halo-Cenpa and 1 µg/ml puromycin for H2B-iRFP. After a week of passaging for puromycin and 2 weeks for geneticin, cells were sorted using a MoFlo flow sorter (Beckman Coulter) so they were haploid and labelled with the relevant mEos3 fluorophore (excitation at 488 nm, emission at 515 nm), as previously described [2, 3].

## Particle trapping experiments


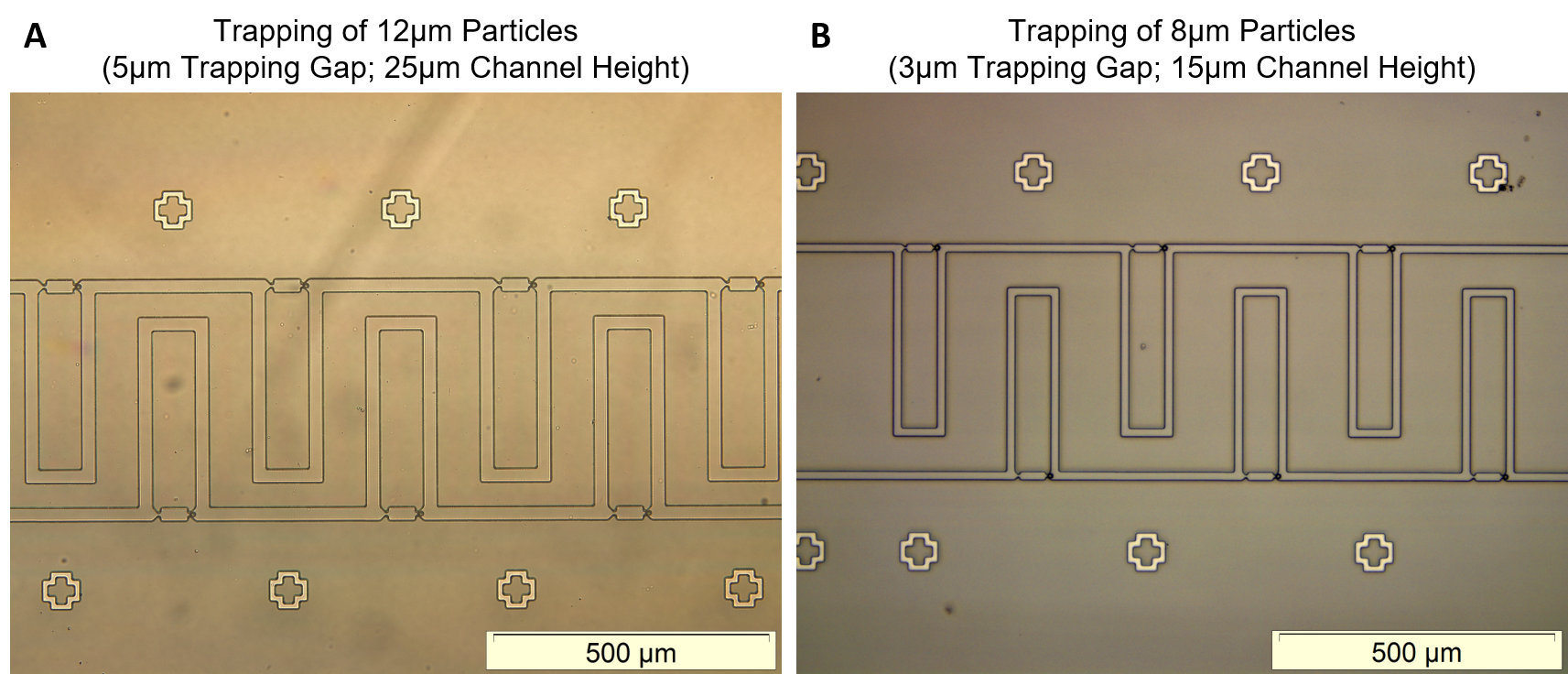


Fig. S4: Examples of single particle trapping. (A) Trapping of 12 µm particles using the device with 5 µm trapping gap and 25 µm channel height. (B) Trapping of 8 µm particles using the device with 3 µm trapping gap and 15 µm channel height.


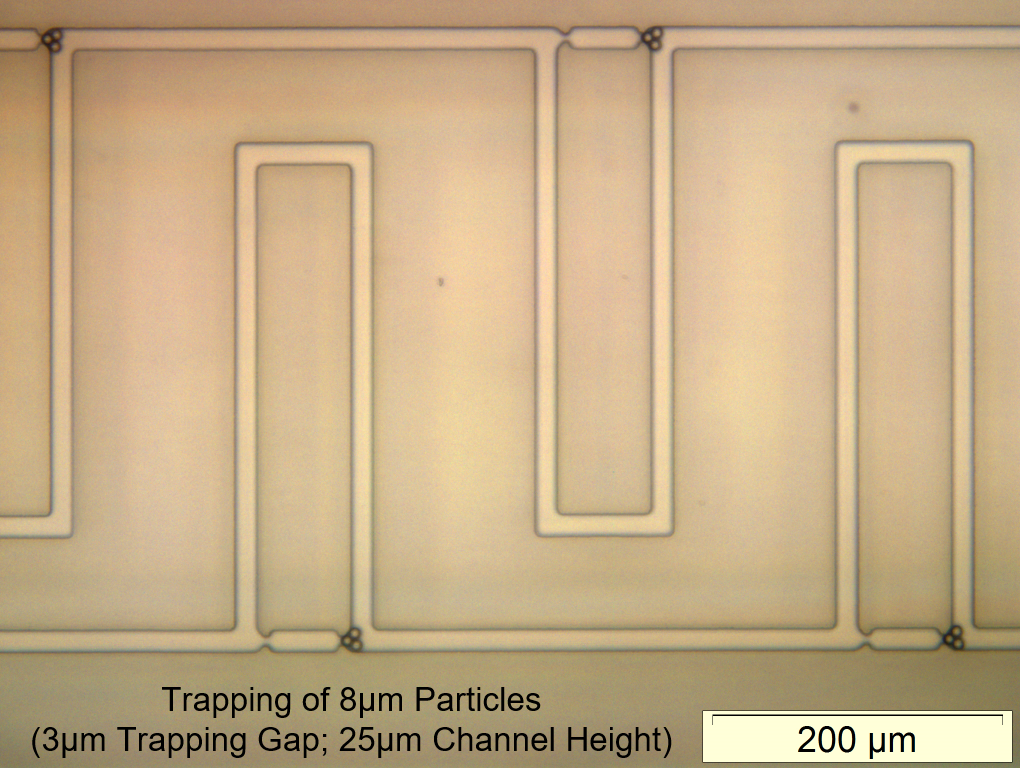


Fig. S5: Example of trapping multiple particles in one trapping site (particle size is 8 µm; trapping gap is 3µm; channel height is 25 µm). The channel height (25 µm) is approximately three times as large as the particle size (8 µm), resulting in multiple particles immobilised in the same trap (in this case three particles per trap).

## Valve testing


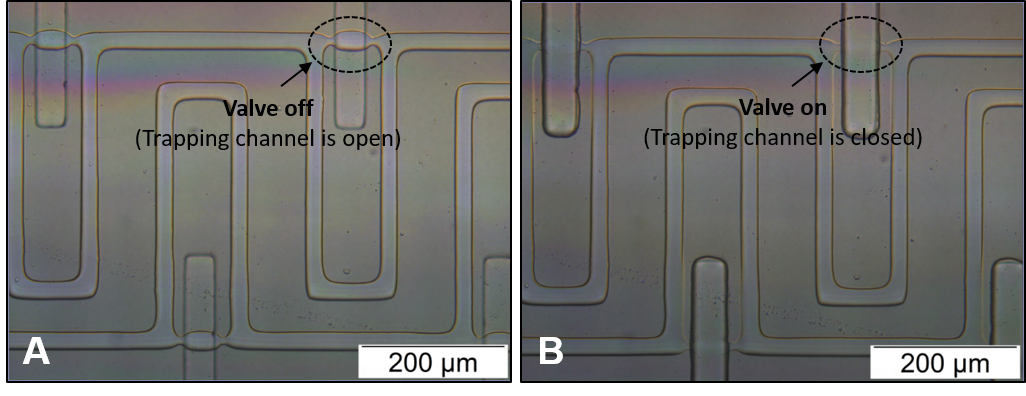


Fig. S6: Examples of deactivated valves (A) and activated valves (B). Control channels are filled with DI water instead of air to prevent air from diffusing through the valve membrane thereby introducing bubbles into the flow layer. Due to the gas permeable properties of PDMS, the dead-end control channels will be depleted of air as the fluid keeps being pushed into the control channels, resulting in a bubble-free fluid-filled control channel. Once the control channels are filled, the on-chip valves could be closed if a further pressure is applied to overcome the PDMS elasticity and the back pressure in flow layer. Valve sealing can be observed at the overlapping region of the trapping channel and the valve channel.


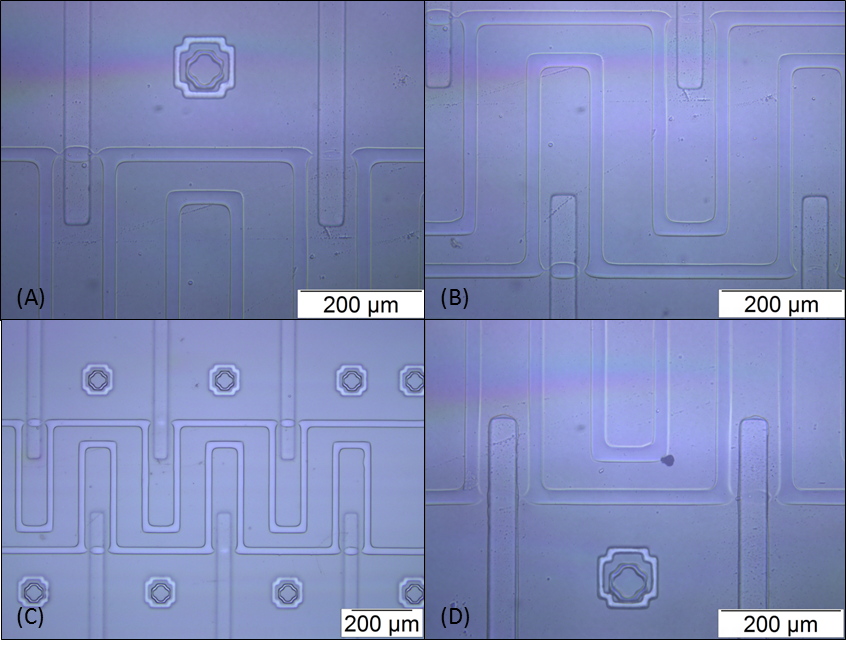


Fig. S7: Examples of valve opening and closing in the same device. (A) The left valve is open while the right one is completely sealed. (B) The first two (from left) valves are open, whereas the other two valves are fully closed. (C) The two valves in the middle are completely closed, while other valves are not activated (opened). (D) The left valve is completely sealed, whereas the right valve is partially closed. A reduced pressure in control channel will close the flow channel partially, reducing the size of the flow path and changing the flow resistance in the flow channel.

## Particle trapping & releasing


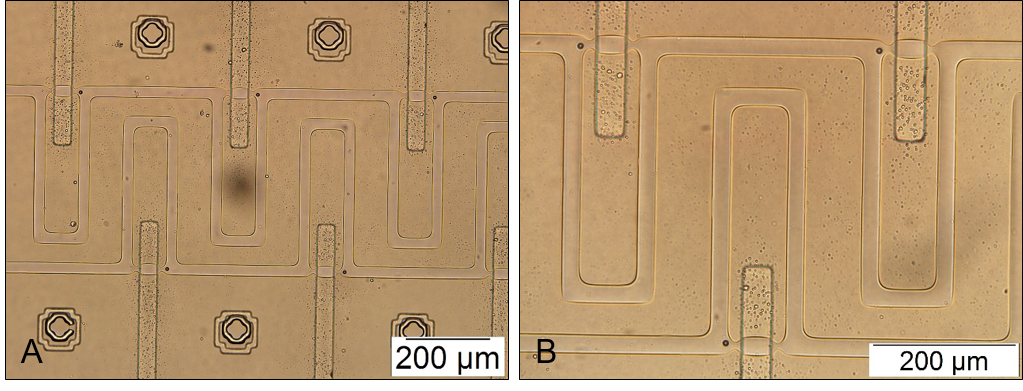


Fig. S8: Examples of single particle trapping. (A) Particle trapping when the flow direction is from right to left. (B) Particle trapping when the flow direction is from left to right.


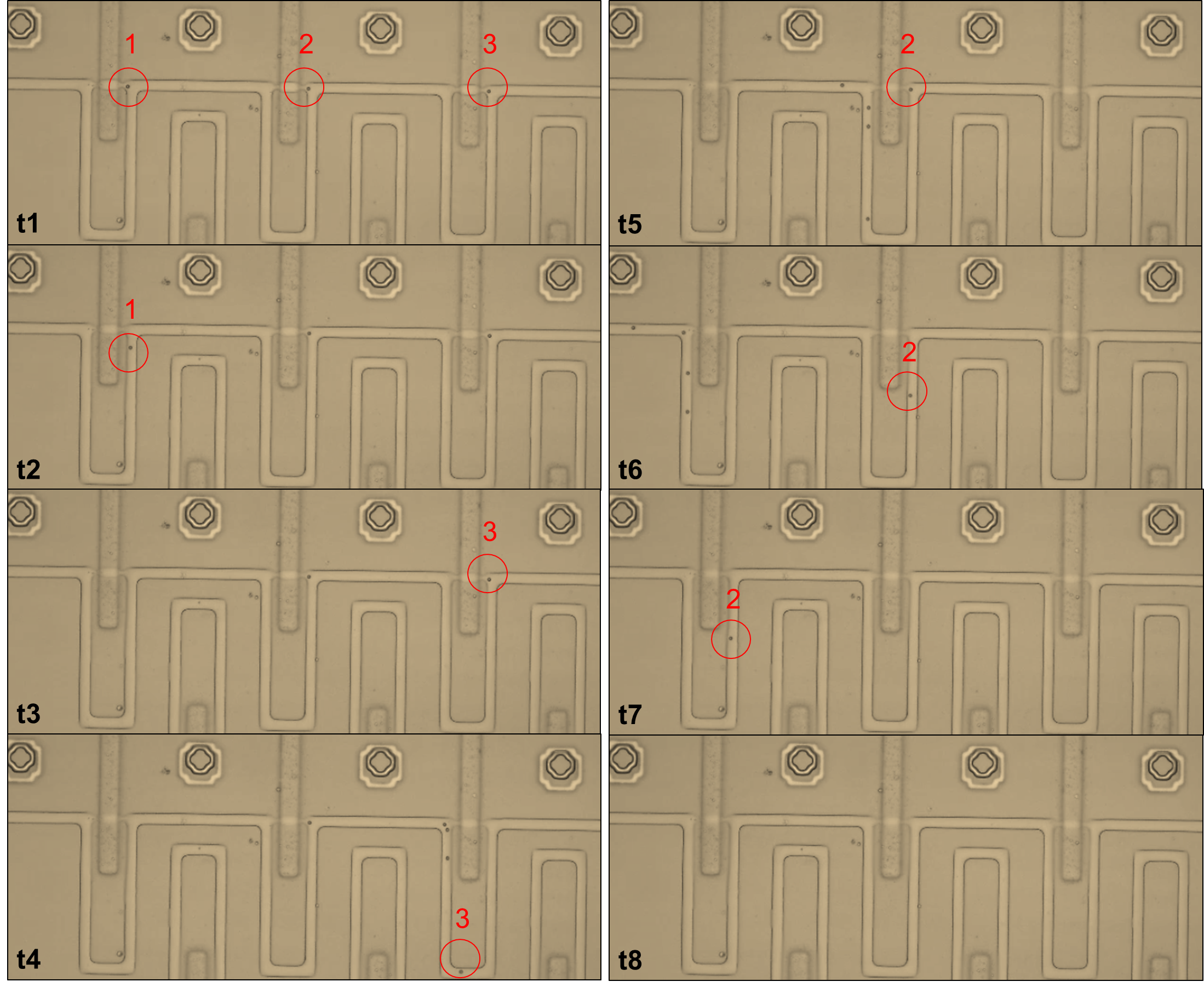


Fig. S9: Time-lapse images of particle release process recorded at different time points. The flow direction is from right to left. i) At time t1 (t=0s; starting point), three particles are trapped, notated as ‘1’, ‘2’ and ‘3’ respectively in the figure. ii) At t2 (t=23s), the left particle is released from the trap by the closing the valve. iii) At t3 (t=25s), the left particle is totally flowing out of the frame being recorded. iv) At t4 (t=56s), the right particle is released from the trap. The other three more particles appeared in the image are actually released from other traps on the right-hand side, which are outside the recorded frame. v) At t5 (t=58s), the released particles keep flowing to the left in the channel. vi) At t6 (t=61s), the middle particle is released from the trap. At the meantime, instead of being trapped, all other particles are flowing bypass the leftmost trap. This is because the left trap has already been closed by the valve and will not be available for trapping anymore. vii) At t7 (t=63s), the particle that was released from the middle trap is bypassing the left trap and continue flowing in the main channel to the left outlet. viii) At t8 (t=65s), all particles are released from their traps and driven out the device.

## Particle trapping test when valve is on


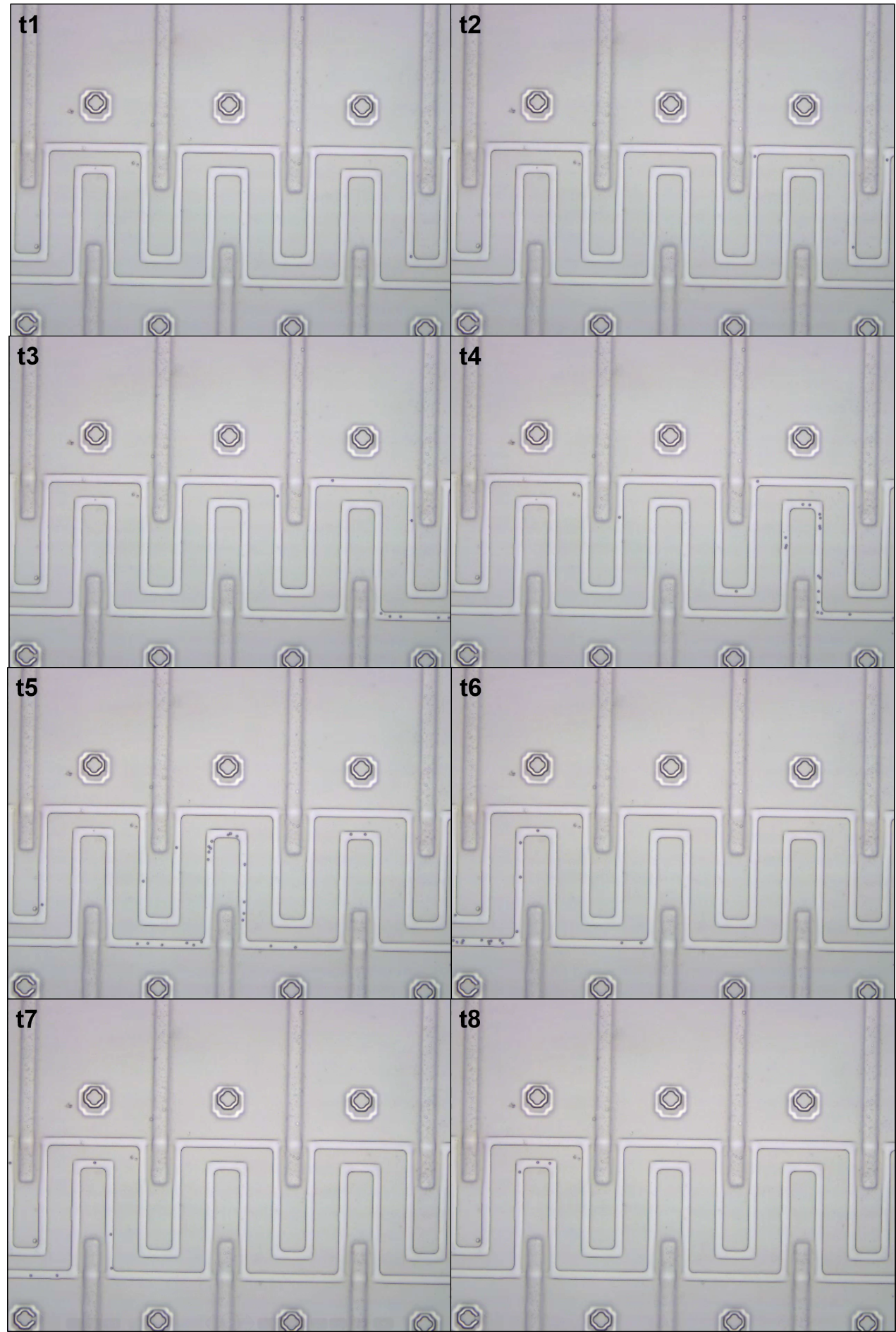


Fig. S10: Particle flow trajectories when valves are activated. The flow direction is from right to left. No particle is observed to be trapped when valves are completely sealed. Timescale: t1=0s (starting point); t2=3s; t3 =7s; t4=9s; t5=15s; t6=21s; t7=22s; t8=23s.

## Precision of single mEos3-tagged Cenpa molecules in PALM microscopy movies


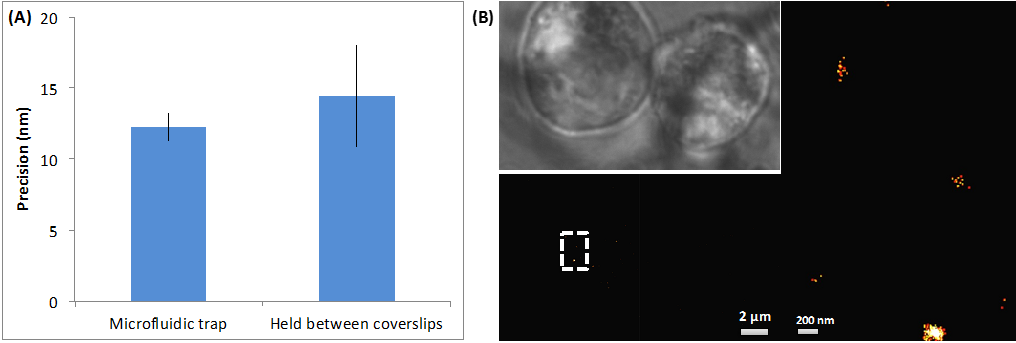


Fig. S11: (A) Average precision of single mEos3-tagged Cenpa molecules imaged using PALM in the trapped microfluidic device compared to those in adherent cells immobilised to the cover slip (n = 3 cells per sample). (B) An example super-resolution image taken of mEos3-tagged Cenpa molecules in adherent cells is provided for reference.

# References

[1] L. Bell, A. Seshia, D. Lando, E. Laue, M. Palayret, S.F. Lee, et al., A microfluidic device for the hydrodynamic immobilisation of living fission yeast cells for super-resolution imaging, Sensors Actuators B: Chem, 192(2014) 36-41.

[2] M. Leeb, A. Wutz, Derivation of haploid embryonic stem cells from mouse embryos, Nature, 479(2011) 131-U64.

[3] M. Palayret, H. Armes, S. Basu, A.T. Watson, A. Herbert, D. Lando, et al., Virtual-'Light-Sheet' Single-Molecule Localisation Microscopy Enables Quantitative Optical Sectioning for Super-Resolution Imaging, PLoS One, 10(2015).

[4] Y. Miyanari, C. Ziegler-Birling, M.E. Torres-Padilla, Live visualization of chromatin dynamics with fluorescent TALEs, Nat Struct Mol Biol, 20(2013) 1321-U252.
